# Changes in Activity of Spinal Postural Networks at Different Time Points After Spinalization

**DOI:** 10.3389/fncel.2019.00387

**Published:** 2019-08-21

**Authors:** Pavel V. Zelenin, Vladimir F. Lyalka, Grigori N. Orlovsky, Tatiana G. Deliagina

**Affiliations:** Department of Neuroscience, Karolinska Institute, Stockholm, Sweden

**Keywords:** spinal cord injury, balance control, postural reflexes, spinal neurons, spinal networks, spasticity

## Abstract

Postural limb reflexes (PLRs) are an essential component of postural corrections. Spinalization leads to disappearance of postural functions (including PLRs). After spinalization, spastic, incorrectly phased motor responses to postural perturbations containing oscillatory EMG bursting gradually develop, suggesting plastic changes in the spinal postural networks. Here, to reveal these plastic changes, rabbits at 3, 7, and 30 days after spinalization at T12 were decerebrated, and responses of spinal interneurons from L5 along with hindlimb muscles EMG responses to postural sensory stimuli, causing PLRs in subjects with intact spinal cord (control), were characterized. Like in control and after acute spinalization, at each of three studied time points after spinalization, neurons responding to postural sensory stimuli were found. Proportion of such neurons during 1st month after spinalization did not reach the control level, and was similar to that observed after acute spinalization. In contrast, their activity (which was significantly decreased after acute spinalization) reached the control value at 3 days after spinalization and remained close to this level during the following month. However, the processing of postural sensory signals, which was severely distorted after acute spinalization, did not recover by 30 days after injury. In addition, we found a significant enhancement of the oscillatory activity in a proportion of the examined neurons, which could contribute to generation of oscillatory EMG bursting. Motor responses to postural stimuli (which were almost absent after acute spinalization) re-appeared at 3 days after spinalization, although they were very weak, irregular, and a half of them was incorrectly phased in relation to postural stimuli. Proportion of correct and incorrect motor responses remained almost the same during the following month, but their amplitude gradually increased. Thus, spinalization triggers two processes of plastic changes in the spinal postural networks: rapid (taking days) restoration of normal activity level in spinal interneurons, and slow (taking months) recovery of motoneuronal excitability. Most likely, recovery of interneuronal activity underlies re-appearance of motor responses to postural stimuli. However, absence of recovery of normal processing of postural sensory signals and enhancement of oscillatory activity of neurons result in abnormal PLRs and loss of postural functions.

## Introduction

Animals and humans maintain the basic body posture due to the activity of the postural control system. Normal operation of this system is important for standing, for keeping balance during locomotion (Horak and Macpherson, 1996; Macpherson et al., 1997a; Orlovsky et al., 1999), as well as for providing postural support for voluntary movements (Massion and Dufosse, 1988). In terrestrial quadrupeds, the postural system responsible for maintenance of the dorsal-side-up trunk orientation is driven mainly by sensory signals from limb mechanoreceptors (Inglis and Macpherson, 1995; Horak and Macpherson, 1996; Deliagina et al., 2000, 2006, 2012; Beloozerova et al., 2003; Stapley and Drew, 2009). It was demonstrated that this system consists of two relatively independent sub-systems controlling orientation of the anterior and posterior parts of the body in the transverse plane, respectively (Beloozerova et al., 2003; Deliagina et al., 2006).

Earlier, in decerebrate rabbits, we characterized postural limb reflexes (PLRs), which in intact animals significantly contribute to postural corrections generated in response to perturbation of the body posture during standing (Musienko et al., 2008, 2010; Deliagina et al., 2012), as well as to keeping balance during walking (Musienko et al., 2014). We demonstrated that though the spinal cord contains neuronal networks generating EMG pattern of PLRs, efficacy of spinal PLRs is low, and thus, contribution of supraspinal signals is important for generation of functional PLRs (Musienko et al., 2010; Deliagina et al., 2014). Two groups of spinal interneurons (F-neurons and E-neurons) contributing to generation of PLRs have been revealed (Hsu et al., 2012; Zelenin et al., 2015). Since PLRs cause a change in activity of limb extensors, and during PLRs F- and E-neurons were activated in-phase and in anti-phase with extensors, it was suggested that some of them are pre-motor interneurons exciting and inhibiting extensor motoneurons, respectively (Zelenin et al., 2015).

Spinalization results in dramatic impairment of the postural system and postural functions practically do not recover with time (Macpherson et al., 1997b; Macpherson and Fung, 1999; Rossignol et al., 1999, 2002; Barbeau et al., 2002; Lyalka et al., 2011; Chvatal et al., 2013). Immediate effect of spinalization is spinal shock, characterized by muscular hypotonus, and absence of most spinal reflexes (Ashby and Verrier, 1975; Brown, 1994; Ditunno et al., 2004). Spinal networks, deprived of supraspinal influences, undergo considerable spontaneous changes over time (Frigon and Rossignol, 2006; Roy and Edgerton, 2012; D’Amico et al., 2014), which result in gradual development of spasticity, characterized by abnormal reflex responses, clonus, spasms and hypertonus (Brown, 1994; Young, 1994; Hultborn, 2003; Hyngstrom et al., 2008; Frigon et al., 2011; Johnson et al., 2013, 2017). In postural functions, spasticity is manifested as incorrect motor responses to posture-related sensory signals and oscillatory EMG activity in limb muscles (Lyalka et al., 2011). The aim of the present study was to reveal the changes in spinal postural networks underlying the development of spasticity.

Recently, we characterized the starting point for development of spasticity that is the state of spinal postural networks in the spinal shock condition (just after acute spinalization) (Zelenin et al., 2013, 2016a). We found a significant decrease in activity of F- and E-neurons, changes in the distribution of F and E-neurons in the spinal gray matter, as well as distortions in processing of posture-related sensory information. It was also demonstrated that acute spinalization causes a decrease in the excitability of spinal motoneurons (Barnes et al., 1962; Walmsley and Tracey, 1983; Johnson et al., 2013). These distortions in operation of postural networks lead to the loss of postural functions observed in subjects in the spinal shock condition.

Here, to reveal plastic changes in spinal postural networks underlying the development of spasticity, rabbits at 3, 7, and 30 days after spinalization at T12 were decerebrated, and responses of spinal interneurons from L5 to stimulation that evoked PLRs in subjects with intact spinal cord, were recorded. The results were compared with control data from animals with intact spinal cord, as well as with data obtained after acute spinalization in our previous studies (Zelenin et al., 2013, 2015, 2016a).

Short accounts of some parts of this study has been published as abstracts (Deliagina et al., 2015; Lyalka et al., 2017).

## Materials and Methods

Experiments were carried out on 16 adult male New Zealand rabbits (weighing 2.5–3.0 kg). All experiments were conducted in accordance with NIH guidelines and were approved by the local ethical committee (Norra Djurförsöksetiska Nämnd) in Stockholm. The data for F-, E-, and non-modulated neurons obtained in experiments were compared with the control data and with data obtained after acute spinalization taken from the database of our earlier studies. The experimental subjects, as well as all methods used in the control study (except for spinalization, Zelenin et al., 2015) and in the study devoted to acute spinalization (Zelenin et al., 2016a) were similar to those used in the present study. The control data for F-, E-, and non-modulated neurons, as well as data devoted to effect of acute spinalization on F-, E-, and non-modulated neurons have been published earlier (Zelenin et al., 2015, 2016a, respectively).

### Surgical Procedures

Each rabbit was subjected to two operations. The first surgery was performed under Hypnorm-midazolam anesthesia, using aseptic procedures. The level of anesthesia was controlled by applying pressure to a paw (to detect limb withdrawal), and by examining the size and reactivity of pupils. In 13 rabbits during first surgery a laminectomy at the T11–T12 level was done, and the dura in the rostral part of the T12 segment was opened. Then, a complete transection of the spinal cord was done under the dissecting microscope by means of a small scalpel. Later, the incision was closed in anatomic layers.

In 3 (*N* = 5), 7 (*N* = 3), and 30 (*N* = 5) days after spinalization, rabbits were taken to acute experiment. For induction of anesthesia, the animal was injected with propofol (average dose, 10 mg/kg, administrated intravenously). Afterward, it was continued on isoflurane (1.5–2.5%) delivered in O_2_. The trachea was cannulated and laminectomy at L5 (exposing the spinal cord for recording of neurons) was performed. To insert the recording microelectrode, small holes (∼1 mm^2^) were made in the dura mater at L5. Bipolar EMG electrodes were inserted bilaterally into two representative extensors: gastrocnemius lateralis (ankle extensor) and vastus lateralis (knee extensor). The rabbit was decerebrated at the precollicular-postmammillary level (Musienko et al., 2008), and then, the anesthesia was discontinued. The rectal temperature and mean blood pressure of the decerebrate preparation during the experiment were kept, respectively, at 37–38°C and at greater than 90 mmHg. Collection of data began 1.5–2 h after decerebration.

In three rabbits during the first surgery chronic implantation of bipolar EMG electrodes was perform bilaterally into *m. gastrocnemius lateralis* (Gast, ankle extensor) and *m. vastus lateralis* (Vast, knee extensor) by using the method described earlier (Lyalka et al., 2011). After complete recovery from the surgery (in 3–4 days), postural responses of the rabbit to tilts were recorded (see below). Then a second surgical operation (that is spinalization at T12 level) was performed. At 3, 7, and 30 days after spinalization, the animal was subjected to tests on the tilting platform.

### Animal Care

After surgery each animal was individually caged and had access to food (dry rabbit food, hay, and carrots) and water. The bottom of the cage was covered by absorbing soft tissue. The animals were monitored attentively. Every 12 h for 48 h after surgery, the rabbits received an analgesic (Temgesic, 0.01 mg/kg sc). In addition, Rimadyl (4 mg/kg sc) was given before surgery and 2 days after surgery to reduce inflammation. The first 2 days after spinalization the animals received 25 ml of Ringer solution (sc) twice a day. In spinal animals the bladder was expressed manually a few times daily, as well as the hindquarters were inspected and cleaned if necessary.

### Experimental Designs

**FIGURE 1:**
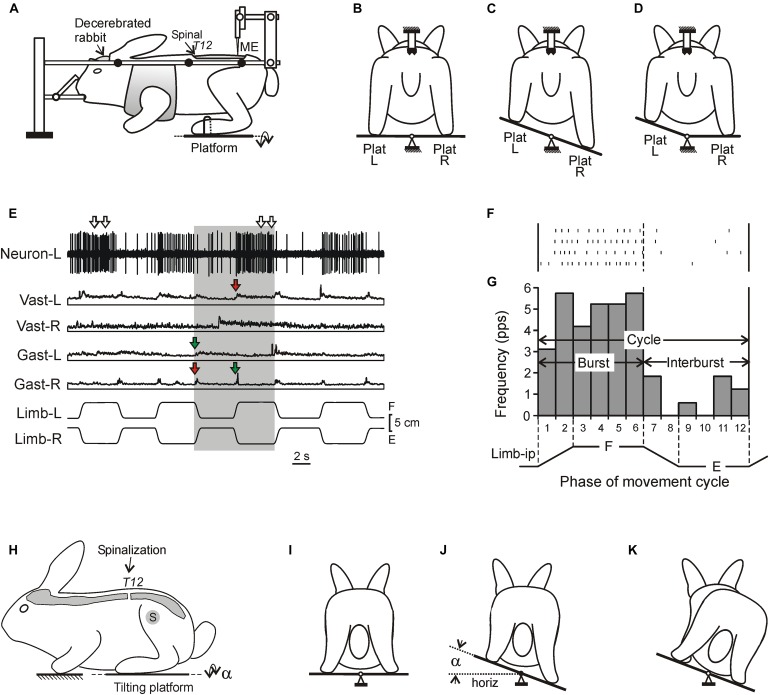
Experimental designs. **(A–D)** A design for acute experiments. The chronic spinalized at T12 level rabbit was decerebrated and fixed in a rigid frame [points of fixation are indicated by black circles in **(A)**]. The whole platform **(C)** or its left or right part [Plat L and Plat R in **(D)**] could be periodically tilted, causing flexion–extension movements [F and E in **(E)**, respectively] of the two limbs (in anti-phase) or one of them, respectively. These movements were monitored by mechanical sensors (Limb-L and Limb-R, respectively). Activity of spinal neurons from L5 was recorded by means of the microelectrode [ME in **(A)**]. **(E)** Responses of a neuron from the left side of the spinal cord (Neuron-L) and electromyographic (EMG) responses in the left and right *m. gastrocnemius lateralis* (Gast-L and Gast-R, respectively) and m. *vastus lateralis* (Vast-L and Vast-R, respectively) to flexion/extension anti-phase movements of the hindlimbs in the rabbit on day 7 after spinalization. Red and green arrows indicate, respectively, residual correctly and incorrectly phased (in relation to the platform tilts), responses in Vast and Gast muscles. White arrows indicate oscillatory bursts in neuronal response. **(F,G)** A raster of responses of the neuron shown in **(E)** in four sequential movement cycles of the ipsilateral limb and a histogram of its spike activity in different phases (1–12) of the cycle of movement (F, flexion; E, extension) of the ipsilateral limb (Limb-ip). The neuron was activated with flexion of the ipsilateral limb (F-neuron). The halves of the cycle with higher (F, bins 1–6) and lower (E, bins 7–12) neuronal activity were designated as “burst” and “interburst” periods, respectively. **(H–K)** Testing of postural reactions to tilts. The animal was standing on two platforms, one under the forelimbs and one under the hindlimbs. Platform under the hindlimbs could be tilted in the transverse plane (α is the platform tilt angle). The sagittal plane of the animal was aligned with the axis of platform rotation. **(J)** Normal postural reaction to tilt in intact rabbit. **(K)** Absence of postural reaction to tilt in spinal rabbit.

**FIGURE 2:**
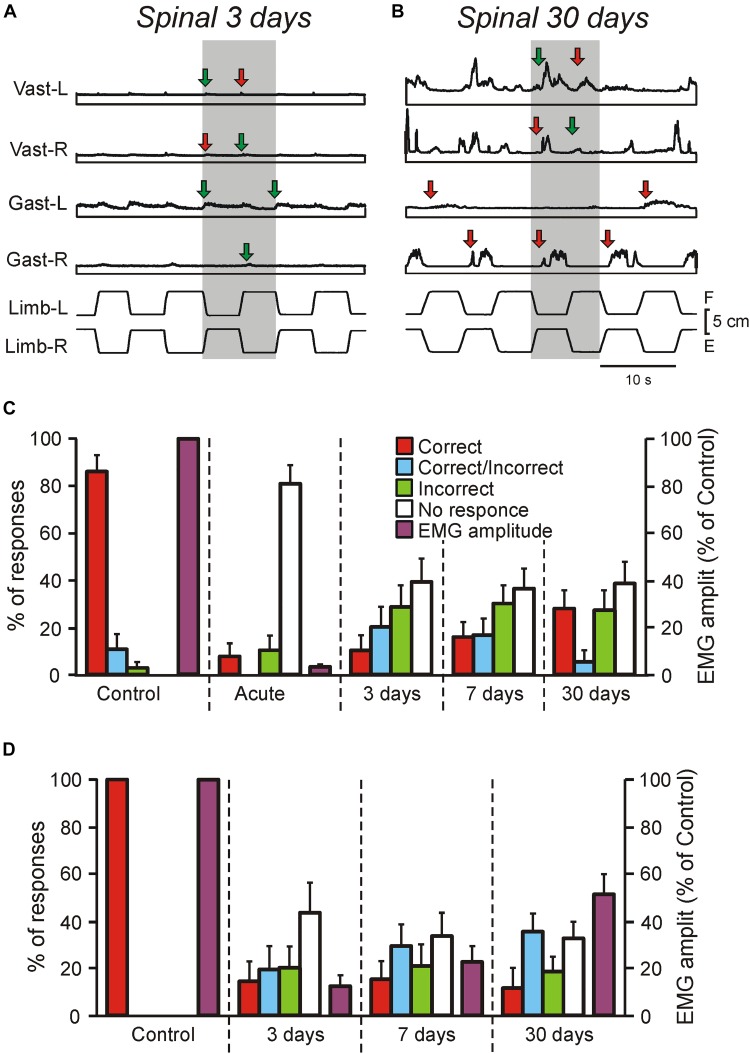
Motor responses of rabbits and decerebrate preparations to tilts in control and at different time points after spinalization. **(A–C)** EMG responses to the whole platform tilts in the rabbit decerebrated on day 3 after spinalization **(A)** and in the rabbit decerebrated on day 30 after spinalization. The EMGs of the following muscles are presented: left (L) and right (R) vastus (Vast), and gastrocnemius (Gast). Red and green arrows in **(A,B)** indicate onsets of correct and incorrect responses, respectively. **(C)** Proportion of different types of EMG responses to the whole platform tilts in Vast and Gast recorded in decerebrate rabbits with intact spinal cord (Control, *N* = 5, *n* = 50), after acute spinalization (Acute, *N* = 5, *n* = 50), at 3 days (*N* = 3, *n* = 18), 7 days (*N* = 3, *n* = 24), and 30 days (*N* = 6, *n* = 34) after spinalization, as well as their amplitude in control and after acute spinalization (*N* = 3, *n* = 18). Correct, activation with ipsi-limb flexion; Incorrect, activation with contra-limb flexion; Correct/Incorrect, activation with both movements; No response, no EMG response to tilt. **(D)** Proportion of different types of EMG responses to the whole platform tilts in Vast and Gast recorded in the same rabbits before spinalization, at 3, 7, and 30 days after spinalization, as well as their amplitude (*N* = 3, *n* = 37, 39, and 40, respectively). Correct, activation with ipsilateral tilt; Incorrect, activation with contralateral tilt; Correct/Incorrect, activation with both ipsi- and contra-tilts; No response, no EMG response to tilt.

The design for acute experiments (Figure 1A) was similar to that described earlier (Musienko et al., 2010; Hsu et al., 2012; Zelenin et al., 2013, 2015). Shortly, the head and spine were fixed, the hindlimbs with configuration similar to that in intact standing rabbit (Beloozerova et al., 2003) were placed on the horizontal platform while the forelimbs were hung in a hammock. The whole platform or its left and right part separately, could be tilted to the left and to the right around the medial axis (Figures 1B–D) with the amplitude ±20°. The platform tilts caused flexion/extension movements at the hindlimb joints and almost vertical displacements of the limb distal point with magnitude ∼5 cm. A trapezoidal time trajectory of the platform tilt causing trapezoid trajectory of foot displacement was used. The duration of the rotation from the right to the left or from the left to the right was ∼1 s and each tilted position was maintained during ∼3–4 s. The angle of the platform tilt was monitored by a mechanical sensor, and after scaling, represented the vertical foot position (Figures 1E, 2A,B). In decerebrate rabbits with intact spinal cord, sensory signals from hindlimbs caused by the platform tilts evoked PLRs: activation of extensors in the flexing limb leading to an increase in its contact force and inactivation of extensors in the extending limb resulting in a decrease in its contact force (Musienko et al., 2010).

Postural test of intact rabbits freely standing on a tilting platform have been described earlier (Deliagina et al., 2000; Beloozerova et al., 2003; Lyalka et al., 2009, 2011). The test did not require a specific training of the animals. In short, the rabbit was placed on two horizontal platforms (one platform supported the forelimbs and another one – the hindlimbs, Figure 1H). The platform supporting the hindlimbs could be tilted in the transverse plane of the animal (angle α, Figures 1H,J). The amplitude and trapezoidal time trajectory of tilting were the same as in experiments on decerebrate rabbits. In intact rabbits the tilt of the platform supporting the hindquarters evoked corrective hindlimb movements stabilizing the dorsal-side-up orientation of the caudal part of the trunk (Figure 1J; Beloozerova et al., 2003), while in spinal rabbits the corrective movements were absent and the trunk followed the platform movement (Figure 1K; Lyalka et al., 2011). During postural test EMGs from selected hindlimb muscles were recorded along with the tilt angle of the platform.

### Recordings and Data Analysis

**FIGURE 3:**
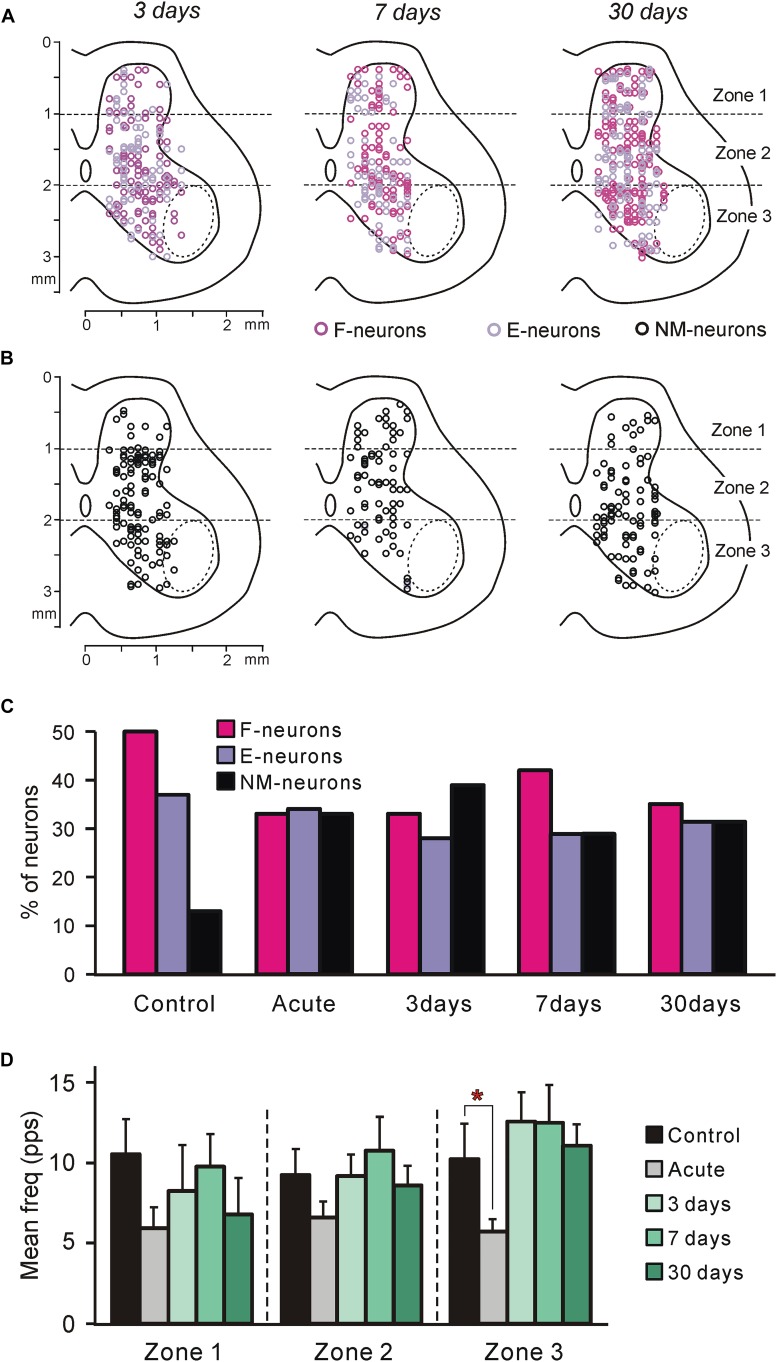
Neurons recorded at different time points after spinalization. **(A,B)** Position of all F- and E-neurons **(A)**, as well as all non-modulated neurons **(B)** on the cross-section of the spinal cord recorded on 3rd, 7th, and 30th day after spinalization. **(C)** Relative number of F-, E-, and non-modulated neurons in control and at different time points after spinalization. The number of F-, E-, and non-modulated neurons, respectively, was *n* = 249, 186, and 64 in control, *n* = 122, 127, and 121 after acute spinalization, *n* = 91, 76, and 108 on day 3, *n* = 98, 68, and 69 on day 7, and *n* = 93, 86, and 86 on day 30 after spinalization. **(D)** Activity of non-modulated spinal neurons in control and at different time points after spinalization. The mean and SEM. values of the mean frequency of non-modulated neurons recorded in control, after acute spinalization (Acute), as well as at 3, 7, and 30 days after spinalization are shown for sub-populations of non-modulated neurons located in different zones (1–3) of the gray matter. The numbers of non-modulated neurons recorded in zones 1–3 in control and after acute spinalization were *n* = 14, 27, 23 and *n* = 18, 44, 59, respectively. The numbers of non-modulated neurons recorded in zones 1–3 in spinal rabbits on 3rd, 7th, and 30th day after spinalization were *n* = 9, 60, 39, *n* = 17, 32, 20, and *n* = 11, 38, 37, respectively. Indication of significance level: ^∗^*p* < 0.05.

Spinal neurons were recorded extracellularly in L5 by varnish-insulated tungsten electrodes (75 μm shaft diameter, 4–7 MΩ impedance; FHC, Bowdoin, ME, United States). We had intention to record spinal interneurons from different areas of the gray matter, therefore the motor nuclei area (indicated by the dotted line in Figures 3A,B) was avoided. Location of recorded neurons was marked on the L5 cross section map by using their lateral and vertical coordinates (Shek et al., 1986; Hsu et al., 2012; Zelenin et al., 2013).

Neuronal activity, EMGs and signals from the mechanical sensors were amplified, digitized (with a sampling frequency of 30, 5, and 1 kHz, respectively) and recorded on a computer. EMGs were rectified and smoothed with the time constant, 100 ms. We used the data acquisition and analysis Power1401/Spike2 system (Cambridge Electronic Design, Cambridge, United Kingdom), which also allowed to perform spike-sorting based on the waveform-matching algorithm (vertical template width 10–20%; maximal vertical scaling to match 0%; minimal percentage of points matching the template 75%). Only neurons with stable spike shape were used for analysis.

Since in the majority of neurons the phase of modulation was determined by the tilt-related sensory input from the ipsilateral limb (Hsu et al., 2012; Zelenin et al., 2015), the activity of each neuron in the movement cycle of the ipsilateral limb was characterized. First, the raster of activity of the neuron in sequential cycles was obtained (Figure 1F). Second, each cycle was divided into 12 bins taking as the cycle onset the onset of the limb flexion. Bins 1–2 and 7–8 corresponded to flexion and extension of the limb, respectively, while bins 3–6 and 9–12, to maintenance of the flexed and extend position, respectively (Figure 1G). Then, the spike frequency in each bin was averaged over the identical bins in all cycles and the phase histogram of activity of the neuron was generated (Figure 1G). The mean frequency in bins 1–6 and in bins 7–12 (corresponding to flexion and extension of the ipsilateral limb, respectively) were calculated and compared. The larger value was termed the burst frequency (F_BURST_), while the smaller – the interburst frequency (F _INTER_) (Figure 1G). A neuron with statistically significant difference between its burst frequency and interburst frequency (two-tailed Student’s *t*-test, *P* < 0.05) was considered as modulated by tilts. In addition, the mean cycle frequency [F _CYCLE_ = (F _INTER_ + F _BURST_)/2] and the depth of modulation (M = F _BURST_ – F _INTER_) were calculated.

To reveal changes in the activity of local neuronal populations in different areas of the gray matter at different time points after spinalization, “heatmaps” for the mean frequency and the depth of modulation in control as well as at each of four time points after spinalization were generated (Supplementary Figure S1). To calculate a value of the parameter in a point with coordinates (*x*,*y*) on the heatmap, values of the parameter for the neurons recorded in close proximity to this point were used. Depending on the distance *d* from recording point to the point (*x*,*y*), these values were weighted [Gaussian weighting *w(d)* = *exp(-d^2^/D^2^)* with the spatial constant *D* = 0.4 mm]. To reveal changes in the parameter, subtraction of the heatmap for control from the corresponding heatmap for a particular time point after spinalization was performed (Figure 5).

To characterize changes in motoneuronal activity (excitability) at different time points after spinalization, the mean value of EMG amplitude was determined during tilts and normalized to the amplitude observed in the same animal before spinalization.

As it was shown earlier (Lyalka et al., 2011) and confirmed in the present study, after spinalization, tilt of the platform evoked EMG bursts, which could be superimposed upon ordinary responses of the muscles. To clarify whether interneurons contribute to generation of these EMG oscillations, the oscillatory activity of F- and E-neurons, as well as non-modulated neurons in control and at different time points after spinalization, was compared. We found that the spiking activity of recorded neurons included not only ordinary responses to tilts, which may contain the dynamic component (higher frequency discharge during tilt) and/or the static component (firing with a constant or slowly decreasing frequency during maintenance of the tilt angle), but sometimes also oscillatory activity (irregular spike bursts with durations approximately 0.25–1 s) superimposed on the ordinary activity. Since it is difficult to discriminate between the burst of activity representing the dynamic component of the response to tilt and the oscillatory burst, to characterize quantitatively the oscillatory component of neuronal activity, we analyzed neuronal activity only during periods when the tilt angle was maintained constant (the plateau phases of tilts). For each plateau we converted the spike sequence into the instantaneous frequency, *f*(*t*), and smoothened it with the sliding averaging (time constant 0.25 s). Then using the built-in software functions, we estimated the ordinary neuronal activity with the best linear fit, *f*_*OR*_(*t*), and calculated the oscillatory component, *f*_*OS*_(*t*), as *f*_*OS*_(*t*) = *f*(*t*) – *f*_*OR*_(*t*). Then we calculated the square root average of *f*_*OS*_(*t*), which reflects the absolute amplitude of oscillatory firing, as well as squared ratio of the square root averages of *f*_*OS*_(*t*) to *f*(*t*), which reflects the power of oscillations. The later shows how large were the oscillations compared to the instantaneous frequency, *f*(*t*). Values of the absolute amplitude of oscillatory firing, as well as values of the power of oscillations obtained in a neuron were then averaged separately for tilts to the right and tilts to the left.

All quantitative data in this study were presented as mean ± SEM. To test the statistical significance of difference between means, two-tailed Student’s *t*-test (*P* < 0.05) was used. To estimate the statistical significance of the effects of spinalization on the proportion of F-, E-, and non-modulated neurons, we used Pearson’s χ^2^ test (*P* < 0.05).

In a proportion of the examined neurons, peripheral receptive fields were studied. To reveal receptive fields, palpation of hindlimb muscle bellies, light brushing of the hairs, pinching the skin by fingers were used. In cases, when a cutaneous input was revealed, the responses from the underlying muscles were ignored.

### Histological Procedures

To verify positions of recording sites on the cross-section of the spinal cord, at the end of each experiment we made reference electrolytic lesions in L5. The piece of the spinal cord containing these lesions as well as the piece with the site of spinalization were fixed with 10% formalin solution, frozen and cut to sections of 30 μm thickness. The sections were stained with Cresyl violet. Locations of recording sites were verified in relation to the reference lesions. Examination of sections from the areas of spinalization has shown that the transection of the spinal cord was complete in all rabbits.

## Results

### Motor Responses to Tilts at Different Time Points After Spinalization

We found that well-coordinated EMG pattern of PLRs observed in decerebrate rabbits with intact spinal cord (activation of extensors in the flexing limb, as well as inactivation of extensors in the extending limb; Musienko et al., 2008, 2010), was absent after spinalization at each of studied time points (Figures 1E, 2A,B). Responses of individual muscles to tilts were poorly coordinated. Each muscle could have four types of activity, as well as spontaneously switch between them: (i) correct response (activation during the ipsi-hindlimb flexion, indicated by red arrows in Figures 1E, 2A,B); (ii) incorrect response (activation during the contra-limb flexion, indicated by green arrows in Figures 1E, 2A,B); (iii) correct/incorrect response (response to both ipsi- and contra-hindlimb flexion); and (iv) no response to tilt. In addition, on day 30 after spinalization, tilt could evoke the repetitive EMG bursts in some muscles superimposed on the ordinary EMG responses (Figure 2B). The amplitude of EMG responses, which was very small on day 3 after spinalization (Figure 2A), gradually increased (Figures 1E, 2B).

As seen in Figure 2C, before spinalization (Control) the overwhelming majority of the EMG responses to tilts were correct, while after acute spinalization (Acute) the responses were absent in 81% of cases and very weak residual responses were observed in 19% of cases (and only 8% of them were correct). However, already on day 3 after spinalization, EMG responses were observed in about 60% of cases and there were no substantial changes in the percent of EMG responses on day 7 and 30. Nevertheless, the percentage of correct responses was low (10% on day 3 and 27% on day 30). Acute spinalization produced a dramatic decrease (to 3.5% of the level observed in the same decerebrate rabbits before spinalization) in the amplitude of EMGs in extensor muscles (Gast and Vast).

To assess excitability of motoneurons (reflected in EMG amplitude), as well as to compare changes of EMG responses to tilts in decerebrate rabbits and in the rabbits with intact brain taking place over time after spinalization, three rabbits with chronically implanted EMG electrodes were tested on the tilting platform before and on 3rd, 7th, and 30th day after spinalization (see section “Materials and Methods,” Figures 1H–K). Their postural reactions were similar to those described in our previous studies (Beloozerova et al., 2003; Lyalka et al., 2009, 2011). Before spinalization, rabbits maintained balance when the platform under their hindlimbs was tilted (Figure 1J). The stereotypic postural responses included extension of the hindlimb (due to activation of its extensor muscles) on the side moving downward, and flexion of the hindlimb (due to reduction in activity of its extensors) on the opposite side. These reciprocal flexion/extension movements of the hindlimbs displaced the caudal part of the trunk in the transverse plane, in the direction opposite to the platform tilt, thus reducing its deviation from dorsal-side-up position.

After spinalization, a well-coordinated EMG pattern observed in intact animals was transformed into weak and poorly coordinated activity of individual muscles. As in decerebrate spinal rabbits, each extensor muscle could spontaneously switch between four types of activity: (i) correct response (activation during the ipsilateral tilt), (ii) incorrect response (activation during the contralateral tilt), (iii) correct/incorrect response (response to both ipsilateral and contralateral tilts), and (iv) no response to tilt. In addition, as in decerebrate rabbits, tilts often evoked repetitive EMG bursts superimposed on the response to tilt.

We found that as in decerebrate rabbits, in chronic spinal rabbits EMG responses were observed in about 60% of cases on day 3 after spinalization and there were no substantial changes in the percent of responses on day 7 and 30 (Figure 2D). Like in decerebrate rabbits, at each time point after spinalization, the minority (about 15%) of responses were correct. The EMG amplitude (which constituted only about 13% of control on day 3 after spinalization) gradually increased over time and reached about 50% of control on day 30. Thus, the level of activity in motoneurons did not return to control level 1 month after spinalization. Due to disintegration of the EMG pattern and a decrease in the response magnitude, at each of three time points after spinalization, corrective hindlimb movements were absent and the body followed the platform movement (Figure 1K).

### Spinal Neurons at Different Time Points After Spinalization

#### Proportion of Different Types of Neurons

In rabbits decerebrated 3 (*N* = 5), 7 (*N* = 3), and 30 (*N* = 5) days after spinalization at T12, 275, 235, and 265 neurons, respectively, were recorded during passive flexion/extension limb movements caused by periodical tilts of the whole platform. They were considered as putative interneurons, since the majority of these neurons were recorded outside the motor nuclei area (Figures 3A,B, dotted line).

To reveal the changes in spinal postural networks taking place during the 1st month after spinalization, the data obtained in the present study, were compared to data from our previous studies obtained on decerebrate rabbits with intact spinal cord (control data, Zelenin et al., 2015) and on decerebrate rabbits after acute spinalization (Zelenin et al., 2016a). In those studies a large samples of neurons (*n* = 499 and *n* = 370, respectively) were analyzed. As in control and after acute spinalization, F-, E-, and non-modulated neurons were found in all rabbits on 3rd, 7th, and 30th day after spinalization. Like in control and after acute spinalization, F-, E-, and non-modulated neurons were distributed over the gray matter and intermixed (Figures 3A,B).

We found that at each of the studied time points after spinalization the relative number of F-neurons was significantly smaller than that in control population (χ^2^ test, *P* < 0.0001, *P* < 0.0001, *P* < 0.05, and *P* < 0.0001, respectively, after acute spinalization, at day 3, 7, and 30 after spinalization; Figure 3C). By contrast, the relative number of non-modulated neurons after acute spinalization, as well as at 3, 7, and 30 days after spinalization, was significantly larger than that in control (χ^2^ test, *P* < 0.0001 at each of four time points; Figure 3C). The relative number of E-neurons after spinalization was rather similar to that in control population, though a small but significant decrease was observed at 3 and 7 days after spinalization (χ^2^ test, *P* < 0.001 and *P* < 0.05, respectively; Figure 3C).

#### Activity of Neurons

##### F-Neurons

**FIGURE 4:**
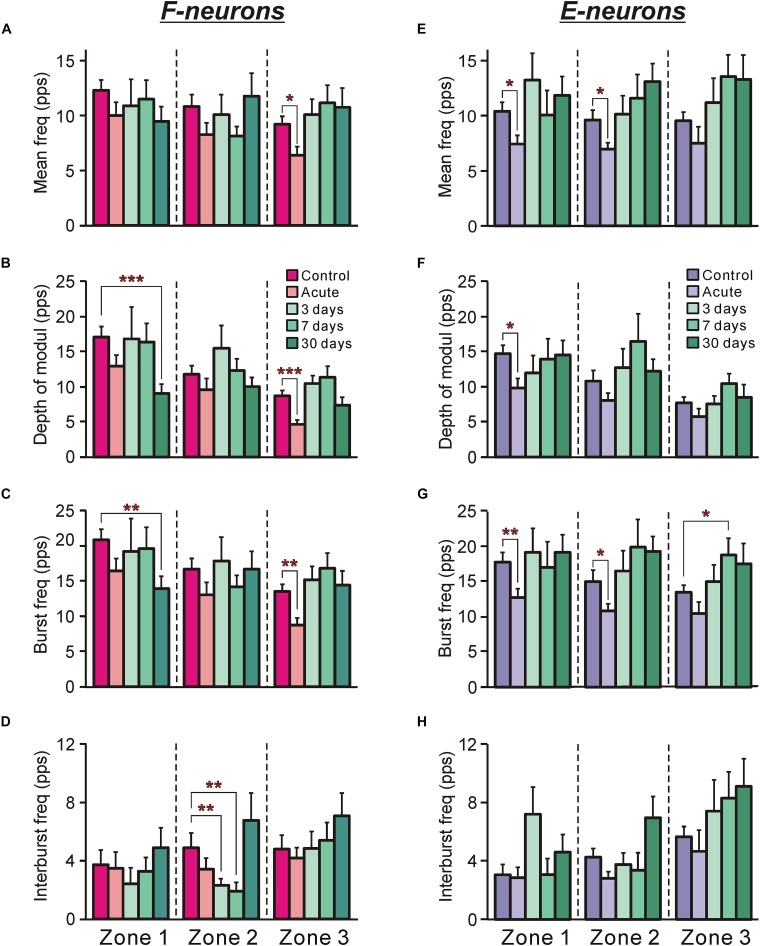
The activity of F-neurons and E-neurons during tilts of the whole platform in control and at different time points after spinalization. The mean and SEM. values of different characteristics of the activity [the mean frequency **(A,E)**, the depth of modulation **(B,F)**, the burst frequency **(C,G)**, and the interburst frequency **(D,H)**] of F-neurons **(A–D)** and E-neurons **(E–H)** in control (Control), after acute spinalization (Acute), and on 3rd, 7th, and 30th days after spinalization (3, 7, and 30 days, respectively). These values are shown for sub-populations of F- and E-neurons located in different zones (1–3) of the gray matter (Figures 3A,B). The numbers of F-neurons recorded in zones 1, 2, 3 in control were *n* = 62, 94, 93, after acute spinalization – *n* = 30, 49, 43, on 3 day – *n* = 15, 36, 40, on 7th day – *n* = 24, 38, 36, on 30th day – *n* = 21, 39, 33. The numbers of E-neurons recorded in zones 1, 2, 3 in control were *n* = 49, 63, 74, after acute spinalization – *n* = 30, 78, 19, on 3 day – *n* = 12, 37, 27, on 7th day – *n* = 17, 18, 33, on 30th day – *n* = 20, 35, 28. Indication of significance level: ^∗^*p* < 0.05, ^∗∗^*p* < 0.01, and ^∗∗∗^*p* < 0.001.

After acute spinalization the main parameters of activity [the mean frequency (Figure 4A), the depth of modulation (Figure 4B) and the burst frequency (Figure 4C)] exhibited a significant decrease, as compared to those in control, in F-neurons located in zone 3. However, already at 3 days after spinalization these parameters reached the control value and remained close to this level at 7 and 30 days (Figures 4A–C). Caused by acute spinalization changes in values of the main parameters of activity of F-neurons located in zones 1 and 2 were insignificant (Figures 4A–D). However, at 30 days after spinalization, F-neurons located in zone 1 exhibited almost 50% decrease in the value of the depth of modulation (9.1 ± 1.3 pps vs. 17.1 ± 1.5 pps in control). It was caused by a significant decrease in the value of their mean burst frequency (14.0 ± 1.6 pps vs. 20.8 ± 1.5 pps in control (Figure 4C). In addition, F-neurons located in zone 2 exhibited a significant decrease (as compared with control) in the mean value of the interburst frequency at 3 and 7 days after spinalization (Figure 4D). However, it returned to the control level at day 30.

To delineate more precisely the areas of the gray matter containing populations of neurons exhibiting a significant change in activity at different time points after spinalization as compared with control, heatmaps (see section “Materials and Methods”) for the mean frequency (Supplementary Figures S1A–F) and the depth of modulation (Supplementary Figures S1F–J) were used.

**FIGURE 5:**
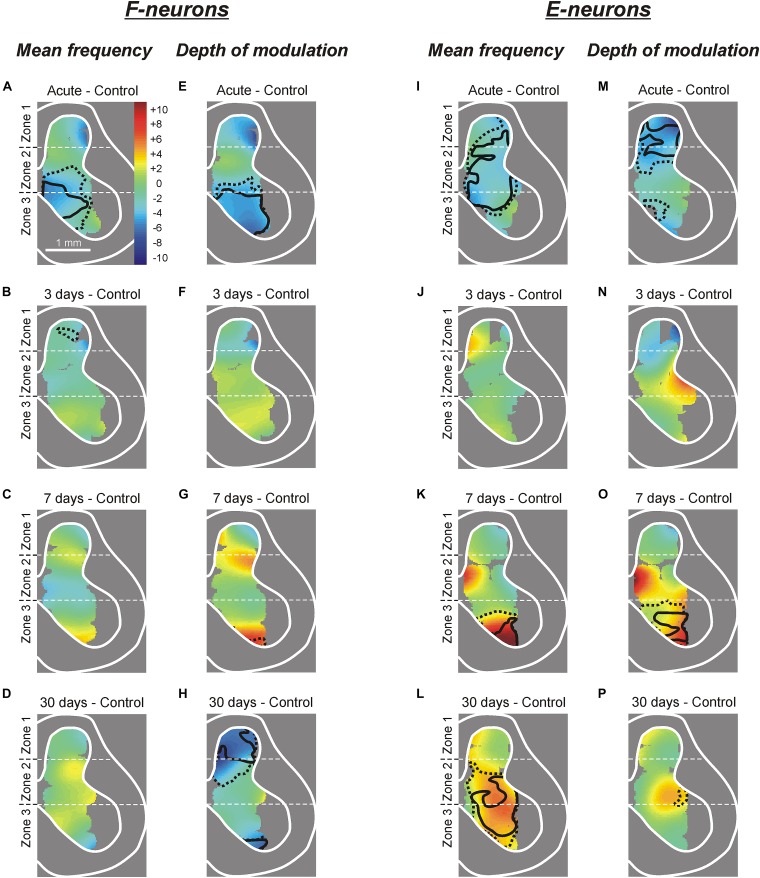
Changes in the mean frequency and in the depth of modulation of local populations of F- and E-neurons at different time points after spinalization. The difference between the averaged distribution of the mean frequency **(A–D,I–L)** and of the depth of modulation **(E–H,M–P)** of F-neurons **(A–H)** and E-neurons **(I–P)** on the cross-section of the spinal cord at a particular time point after spinalization and the corresponding distribution in control (subtraction of Control from corresponding spinal). Averaged distributions of the mean frequency and depth of modulation for F- and E-neurons in control, after acute spinalization, as well as on 3rd, 7th, and 30th day after spinalization are presented as heatmaps in Supplementary Figure S1.

Acute spinalization caused a significant reduction (by 2–6 pps) in the mean frequency of neuronal populations located in the ventral half of zone 2 and the medial part of zone 3 (delineated by solid and hatched lines for *p* = 0.01 and *p* = 0.05, respectively, on subtraction Control from Acute in Figure 5A). All differences in activity of local populations of neurons, observed in 3, 7, and 30 days after spinalization (except for the difference in a small area in the dorsal horn at day 3, Figure 5B), were insignificant as compared with control. Thus, in general, the control value of the mean frequency was restored at 3 days after spinalization and remained at control level during the following 27 days.

Acute spinalization led to a significant decrease in the depth of modulation of the F-neurons located in the ventro-medial part of the ventral horn (Figure 5E). At 3 and 7 days after spinalization, small differences in the depth of modulation of neuronal populations located in different areas of the gray matter, as compared with control were insignificant. Thus, in general, the control value of the depth of modulation was restored at 3 days after spinalization and remained at control level during the following 4 days. However, at 30 days after spinalization we found a significant reduction in the depth of modulation of neuronal populations located in the dorsal horn and in the most ventral part of the zone 3 (Figure 5H).

##### E-Neurons

After acute spinalization the decrease in most parameters of activity was statistically significant in populations of E-neurons located in zones 1 and 2 (Figures 4E–H). We found that at 3 days after spinalization the values of all parameters of activity in zone 1 and zone 2 populations of E-neurons returned to the control level, and did not differ from control level at 7 and 30 days after spinalization (Figures 4E–H). In contrast to F-neurons, after acute spinalization the main parameters of activity of E-neurons located in zone 3 did not differ from those in control. They also were similar to control at 3, 7, and 30 days after spinalization (except for the mean burst frequency at day 7 that was significantly higher than in control, Figure 4G).

Acute spinalization evoked a significant reduction in the mean frequency of E-neurons located in the dorsal part of the ventral horn, in the intermediate area and in the ventro-lateral part of the dorsal horn (Figure 5I), while the depth of modulation was significantly reduced in the population located in the dorsal horn (Figure 5M). Like in F-neurons, at 3 days after spinalization both parameters of activity in populations of E-neurons located in different parts of the gray matter (including those which exhibited a significant reduction of these parameters after acute spinalization) did not differ significantly from control (Figures 5J,N). While at 7 days after spinalization both the mean frequency and the depth of modulation in populations of F-neurons located in different parts of the gray matter did not differ significantly from control (Figures 5C,G, respectively), in populations of E-neurons some significant dynamic changes in activity were observed. Thus, at 7 days after spinalization, both the mean frequency and the depth of modulation in the population of E-neurons located in ventral half of the ventral horn significantly exceeded the control level (Figures 5K,O, respectively). At 30 days after spinalization, the area of the gray matter containing populations of E-neurons exhibiting the mean frequency significantly higher than that in control expanded and occupied almost whole zone 3 and ventral three quarters of zone 2 (Figure 5L). By contrast, the depth of modulation significantly increased (as compared with control) in populations of E-neurons located in the ventral part of zone 3 at 7 days after spinalization (Figure 5O), returned to control level at 30 days after spinalization (Figure 5P).

##### Non-modulated neurons

After acute spinalization, a significant decrease in the mean frequency was observed in population of non-modulated neurons located in zone 3 (Figure 3D). At 3 days after spinalization it returned to control level and was similar to control at 7 and 30 days after spinalization (Figure 3D). After acute spinalization as well as at 3, 7, and 30 days after spinalization, the mean frequency of non-modulated neurons located in zones 1 and 2 did not differ significantly from that in control.

Thus, we found that activity of F-neurons located in the ventral horn, and E-neurons located in the dorsal horn, which was significantly decreased after acute spinalization, returned to control level at day 3 after spinalization. By contrast, F-neurons located in the dorsal horn and E-neurons located in the ventral horn, which activity was not affected by acute spinalization, exhibited significant changes in activity at later time points after spinalization (e.g., in F-neurons, a significant decrease in the mean burst frequency and depth of modulation was observed at day 30 after spinalization, while in E-neurons, a significant increase in the mean cycle frequency and depth of modulation was found at day 7 after spinalization).

### Processing of Tilt-Related Sensory Information

Modulation of neuronal activity caused by tilts is determined by somatosensory inputs from limbs. To characterize the changes in the processing of tilt-related sensory information at different time points after spinalization, in animals at 3rd, 7th, and 30th day after spinalization, respectively, in 133, 147, and 157 neurons, in addition to responses to the whole platform tilts, we recorded responses to tilts of the right and left part of the platform (Figure 1D). The results were compared to control and to those obtained after acute spinalization.

#### Sources of Modulation of F- and E-Neurons

Tilts of either the right or left part of the platform, allowed to reveal the sources of tilt-related sensory input to individual neurons. As in control (Zelenin et al., 2015) and after acute spinalization (Zelenin et al., 2016a), in animals at 3rd, 7th, and 30th day after spinalization, four types of neurons were found. Type 1 and Type 2 neurons received tilt-related sensory input from ipsilateral limb only and from contralateral limb only, respectively (Supplementary Figures S2A–F, respectively). By contrast, Type 3 and Type 4 neurons received sensory inputs from both hindlimbs. However, Type 3 neurons were activated by ipsilateral limb flexion and contralateral limb extension, or ipsilateral limb extension and contralateral limb flexion (Supplementary Figures S2G–I), while Type 4, by flexion of each of hindlimbs (Supplementary Figures S2J–L), or by extension of each of hindlimbs.

**FIGURE 6:**
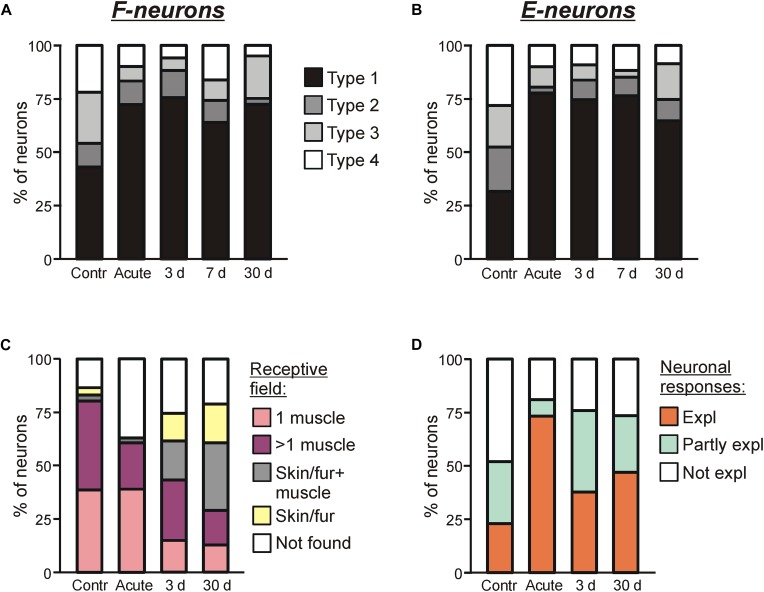
Sources of modulation and receptive fields of F- and E-neurons in control and at different time points after spinalization. Percentage of F-neurons **(A)** and E-neurons **(B)** receiving different combinations of tilt-related somatosensory inputs from the limbs (Types 1–4) in control (*Contr*), after acute spinalization (*Acute*) and on 3rd day (3 days), 7th day (7 days), 30th day (30 days) after spinalization. See text for explanation. **(C)** Proportion of neurons receiving sensory inputs from different sources, i.e., from receptors of only one muscle (*1 muscle*), from receptors of more than one muscle (>*1 muscle*), from cutaneous and muscle receptors (*Skin/fur* + *muscle*), from cutaneous receptors only (*Skin/fur*), and with no receptive field found (*Not found*) in control, after acute spinalization, on 3rd and 30th day after spinalization. See text for explanation. **(D)** Proportion of neurons in which response to tilts could be completely explained (*Expl*), partly explained (*Partly expl*) and could not be explained (*Not expl*) by input from their receptive field, in control, after acute spinalization, on 3rd and 30th day after spinalization.

We found that at each time point after spinalization about twofold increase in relative number of both Type 1 F-neurons (Figure 6A) and Type 1 E-neurons (Figure 6B) was observed. They constituted 73% of F-neurons after acute spinalization, and 75, 64, and 73% at 3, 7, and 30 days after spinalization, respectively, vs. 43% in control, and 78% of E-neurons after acute spinalization, and 75, 77, and 65% at 3, 7, and 30 days after spinalization, respectively, vs. 32% in control. Correspondingly, the proportion of neurons with input from the contralateral hindlimb (Types 2–4), decreased (after acute spinalization, at 3, 7, and 30 days after spinalization, respectively, 28, 25, 36, and 27% of F-neurons vs. 57% in control, and 22, 26, 23, and 35% of E-neurons vs. 68% in control). These changes in proportions of Type 1 neurons and Types 2–4 neurons were significant both for F- and E-groups (χ^2^ test, *P* < 0.0001, except for F-neurons on 7th day after spinalization, which had lower significance level, *P* = 0.001).

#### Efficacy of Sensory Inputs to F- and E-Neurons From Different Limbs

**FIGURE 7:**
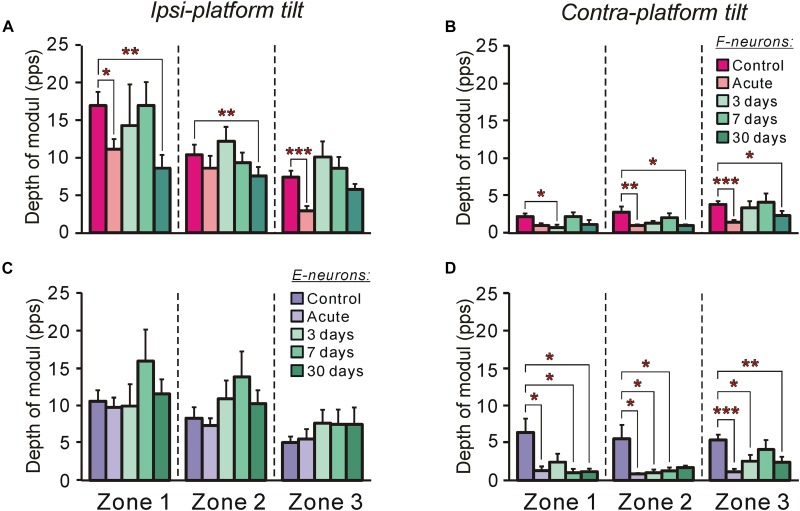
The efficacy of tilt-related sensory inputs from the ipsilateral and contralateral limbs to F- and to E-neurons in control and at different time points after spinalization. **(A,B)** The mean and SEM. values of the depth of modulation of F-neurons recorded in control, after acute spinalization and on 3rd day, 7th day, and 30th day after spinalization during tilts of the ipsilateral **(A)** and contralateral **(B)** limbs. The numbers of F-neurons from zones 1, 2, 3 subjected to these tests were: in control animals – *n* = 42, 62, 71, respectively; in animals after acute spinalization – *n* = 28, 41, 33, respectively; in animals on 3rd day after spinalization – *n* = 12, 24, 31, respectively; in animals on 7th day after spinalization – *n* = 23, 32, 31, respectively; in animals on 30th day after spinalization – *n* = 18, 35, 31, respectively. **(C,D)** The mean and SEM. values of the depth of modulation of E-neurons recorded in control, after acute spinalization and on 3rd day, 7th day, and 30th day after spinalization during tilts of the ipsilateral **(C)** and contralateral **(D)** limbs. The numbers of E-neurons from zones 1, 2, 3 subjected to these tests were: in control animals – *n* = 32, 45, 55, respectively; in animals after acute spinalization – *n* = 25, 67, 17, respectively; in animals on 3rd day after spinalization – *n* = 8, 34, 24, respectively; in animals on 7th day after spinalization – *n* = 16, 16, 29, respectively; in animals on 30th day after spinalization – *n* = 20, 30, 23, respectively. Designations are the same as in Figure 4. Indication of significance level: ^∗^*p* < 0.05, ^∗∗^*p* < 0.01, and ^∗∗∗^*p* < 0.001.

The efficacy of sensory input from a particular limb to the neuron is reflected in its activity modulation depth caused by the tilts of the platform under the limb. In control, the depth of modulation averaged over F-neurons located in each of three zones was much larger during tilts of the ipsilateral platform (Figure 7A) than during tilts of the contralateral platform (Figure 7B). In both cases, in F-neurons located in each of three zones of the gray matter (except for zone 1 in case of contralateral platform tilt) a similar tendency in the changes of this parameter was observed. The value of the depth of modulation, which was substantially decreased after acute spinalization, returned to control level on 3rd day and was maintained on this level on 7th day after spinalization. However, on 30th day after spinalization it was substantially reduced as compared with control and had value similar to that observed after acute spinalization (Figures 7A,B).

In contrast to F-neurons, in control, the depth of modulation averaged over E-neurons located in each of three zones was similar during tilts of the ipsilateral platform and during tilts of the contralateral platform (compare corresponding values in Figures 7C,D). The acute spinalization caused a dramatic reduction (almost to zero) of the depth of modulation caused by tilts of the contralateral limb and it remained almost at the same level on 3rd, 7th, and 30th day after spinalization (Figure 7D). By contrast, the depth of modulation of averaged over E-neurons located in each of three zones caused by tilts of the ipsilateral limb was not affected by acute spinalization and did not differ significantly from control level on 3rd, 7th, and 30th day after spinalization (Figure 7C).

#### Relation Between Responses to Tilts and Receptive Fields of Neurons

On 3rd and 30th day after spinalization, somatosensory receptive fields were found, respectively, in 98 out of 131 and 108 out of 137 tested modulated neurons. The proportion of such neurons at each of these two time points after spinalization was slightly smaller than that in control, but slightly higher than that after acute spinalization (75% on 3rd day and 79% on 30th day vs. 86% in control and 63% after acute spinalization; χ^2^ test, *p* = 0.02, and *p* = 0.002) (Figure 6C).

The relative numbers of neurons with different types of receptive fields observed on 3rd and 30th days after spinalization differed from those observed in control and after acute spinalization (Figure 6C). Thus, in control and after acute spinalization, the majority of neurons (81 and 61%, respectively) had “deep” receptive fields (i.e., the neurons responded to palpation of muscles only), while neurons responding to stimulation of fur or/and skin constituted the minority (6 and 2%, respectively). In contrast, on 3rd and 30th day after spinalization, neurons with “deep” receptor fields represented the minority (57 out of 131, 43%, and 29 out of 137, 40%, respectively), while substantial number of neurons responded to stimulation of fur or/and skin (41 out of 131, 31% and 68 out of 137, 50%). On 3rd and 30th day after spinalization, more than a twofold decrease in the percentage of neurons with deep receptive fields from one muscle as compared with those observed in control and after acute spinalization was revealed (respectively, 15 and 13% vs. 39% in control and 39% after acute spinalization; χ^2^ test, *p* < 0.0001). Also, on 3rd and 30th day after spinalization, a significant decrease in the proportion of neurons in which receptive fields were not found as compared with that after acute spinalization was observed (respectively, 25 and 21% vs. 37%; χ^2^ test, *p* = 0.02, and *p* = 0.002).

For 42 and 34 modulated neurons with deep receptive fields recorded on 3rd and 30th day after spinalization, sensory signals from the receptive field of a neuron presumably caused by tilts were compared with responses of this neuron to tilts. It has been suggested that the tilt of the platform activates load and stretch receptors in flexors and extensors of extending and flexing limb, respectively. We found that on 3rd and 30th day after spinalization, in 38 and 47% of neurons, respectively, the response to tilts could be explained by sensory inputs from the receptive field (*Expl* in Figure 6D, 3 and 30 days, respectively), while in 24 and 26% of neurons, respectively, it could not be explained (*Not expl* in Figure 6D, 3 and 30 days, respectively). Finally, 38% of neurons recorded on 3rd day after spinalization and 27% of neurons recorded on 30th day after spinalization had sensory inputs that could explain responses to tilts as well as mismatching inputs, e.g., excitatory inputs from the antagonistic muscles of one limb (*Partly expl* in Figure 6D, 3 and 30 days, respectively). As seen in Figure 6D, on 3rd and 30th day after spinalization, almost twofold decrease in the proportion of neurons in which input from the receptive field could explain the response to tilts, and almost fivefold increase in the proportion of neurons in which response could be partly explained by input from the receptive field as compared to that after acute spinalization were observed (could explain responses: 38% on 3rd day and 27% on 30th day vs. 73% after acute spinalization; could partly explain responses: 38% on 3rd days and 27% on 30th day vs. 8% after acute spinalization). As compared to control, almost twofold decrease in the proportion of neurons, in which response could not be explained by input from the receptive field, observed after acute spinalization was maintained on 3rd and 30th day after spinalization (18% after acute spinalization, 24% on 3rd day, and 26% of the 30th day vs. 48% in control).

Thus, we found severe distortions (as compared with control) in processing of tilt-related sensory signals at each of four time points after spinalization. They were manifested in a change in the source of modulation of F-and E-neurons, in some changes in the efficacy of tilt-related sensory inputs from limbs to these neurons as well as in changes of their receptive fields.

### Oscillatory Activity of Neurons

As it was mentioned above, at later time points after spinalization, tilting often evoked EMG bursts in the hindlimb muscles, which could be superimposed on the ordinary EMG responses (Figure 2B). Bursting (that we term here “oscillatory activity”) caused by tilts was also often observed in activity of recorded neurons (indicated with white arrows in Figure 1E). To characterize this activity quantitatively, we used two parameters: the absolute amplitude of oscillatory activity, and the relative power of the oscillatory signal (see section “Materials and Methods” for details).

**FIGURE 8:**
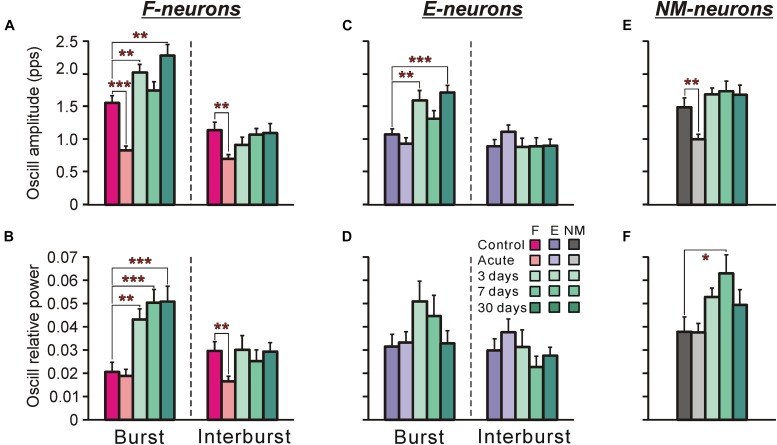
Oscillatory component in activity of F-, E-, and non-modulated neurons during tilts of the whole platform in control and at different time points after spinalization. The mean and SE values of the amplitude of the oscillatory component of activity **(A,C,E)** and the relative power of the oscillatory componet **(B,D,F)** of F-neurons **(A,B)**, E-neurons **(C,D)**, and non-modulated (NM) neurons **(E,F)** in control (*Control*), after acute spinalization (*Acute*), and on 3rd, 7th, and 30th days after spinalization (3, 7, and 30 days, respectively). Designations and numbers of neurons are the same as in Figure 4. Indication of significance level: ^∗^*p* < 0.05, ^∗∗^*p* < 0.01, and ^∗∗∗^*p* < 0.001.

Figures 8A–F show the means ± SEM values of the absolute amplitude of oscillatory activity (Figures 8A,C,E) and the relative power of the oscillatory signal (Figures 8B,D,F) for F-neurons (Figures 8A,B) and E-neurons (Figures 8C,D) calculated separately for the burst and interburst phases of the tilt cycle, as well as for non-modulated neurons (Figures 8E,F). As one can see, some oscillatory activity was exhibited by neurons even in control. Acute spinalization either did not change this activity or resulted in its decrease, which could be seen in a significant reduction of the amplitude of oscillations in F-neurons and in non-modulated neurons (Figures 8A,E, respectively). Since the ordinary responses to tilts were also weakened after acute spinalization, the relative power of oscillatory activity remained the same as in control except for the interbursts in F-neurons (Figure 8B). At 3 days after spinalization an increase in the oscillatory activity of all three groups of neurons was observed. The amplitude of oscillations became significantly higher than in control during bursts of F- and E-neurons, while during interburst phases it returned to the control level (Figures 8A,C). In non-modulated neurons, the amplitude of oscillations also returned to the control level on day 3 after spinalization (Figure 8E). In general, on day 7 as well as on day 30, the amplitude of oscillations in each of three groups of neurons was similar to that observed on day 3.

We found also that in F-neurons, more than twofold increase in the relative power of oscillations during bursts was observed (Figure 8B) from day 3 after spinalization, suggesting that the oscillatory activity constituted substantially larger part of the entire signal, as compared with control. In E-neurons and non-modulated neurons, the average increase in the relative power was not statistically significant, except for the non-modulated neurons at 7 days after spinalization (Figures 8D,F).

To conclude, we found a dramatic increase in oscillatory activity of F-neurons in spinal animals, which could contribute to generation of EMG bursting.

## Discussion

In the present study, we characterized the activity of spinal interneurons of postural networks, and the processing of posture-related sensory signals at three time points after spinalization in rabbits. Comparison of these data with the data obtained in our earlier studies on rabbits with intact spinal cord (control; Zelenin et al., 2015) and on rabbits after acute spinalization (Zelenin et al., 2013, 2016a) allowed us to characterize the changes in the spinal postural networks taking place over time after spinalization and underlying development of spasticity.

As in control and after acute spinalization, on day 3, day 7, and day 30 after spinalization we found three groups of neurons (F-neurons, E-neurons, and non-modulated neurons).

It was demonstrated with the method of reversible spinalization (Zelenin et al., 2013), that disappearance of supraspinal drive did not change the phase of modulation in most F- and E-neurons (though caused a significant decrease in their activity), while some of modulated neurons became non-modulated or completely inactivated. It was suggested that F- and E-neurons recorded after acute spinalization are elements of spinal postural networks (Musienko et al., 2010) contributing to generation of PLRs in animals with intact spinal cord (Zelenin et al., 2016a). Since the relative number of F-, E-, and non-modulated neurons revealed on day 3, 7, and 30 after spinalization was similar to that observed after acute spinalization, one can assume that the majority of F- and E-neurons recorded at these time points after spinalization are elements of the same network that generates PLRs in animals with intact spinal cord.

After spinalization, the relative number of F-neurons significantly decreased, while the number of non-modulated neurons increased as compared with control. The increase in the number of non-modulated neurons could be explained by the fact that a part of neurons modulated before spinalization, after spinalization became non-modulated, while the decrease in the number of F-neurons – by a predominance of F-neurons in the population of modulated neurons completely inactivated by spinalization (Zelenin et al., 2013).

We found that as in subjects with intact spinal cord, at each time point after spinalization F- and E-neurons were evenly distributed across the gray matter and intermixed. This could be explain by the fact that interneurons with inputs from group I and group II afferents from the limbs muscles (which transmit tilt-related sensory signals causing modulation of F- and E- neurons) were found in each of three zones of the gray matter (Bannatyne et al., 2003, 2006, 2009; Jankowska and Edgley, 2010). However, elimination of supraspinal drive caused by acute spinalization affected significantly only specific populations of F- and E-neurons (F-neurons within zone 3 and E-neurons within zones 1 and 2) (Zelenin et al., 2016a). Interneurons with inputs from group I and II afferents and from rubrospinal, reticulospinal and vestibulospinal systems were revealed in laminae VII (located in zones 2 and 3) and laminae VIII (located in Zone 3) (Bannatyne et al., 2003, 2009; Jankowska and Edgley, 2010). Thus, probably the main reason for the decrease in activity of E-neurons in zone 2 and F-neurons in zone 3 is the loss of supraspinal inputs. However, the reason for the significant decrease in activity of E-neurons located in zone 1 is not clear, since revealed in this zone interneurons with input from group II muscle afferents receive corticospinal drive (Bannatyne et al., 2006; Jankowska and Edgley, 2010) which is inactive in decerebrate subject. As the majority of almost completely inactivated by reversible spinalization neurons were F-neurons located in zone 3 (Zelenin et al., 2013), it was suggested that inactivation of premotor F-neurons located in zone 3 substantially contribute to disappearance of PLRs at acute stage of spinal cord injury (Zelenin et al., 2013, 2016a,b). Such pre-motor interneurons with supraspinal inputs and with inputs from group I and II afferents were found in zone 3 (Cavallari et al., 1987; Jankowska et al., 2005, 2009; Bannatyne et al., 2006, 2009; Jankowska, 2008; Stecina et al., 2008; Jankowska and Edgley, 2010; Gosgnach et al., 2017).

Surprisingly, we found that all activity parameters of spinal neurons (mean frequency, burst and interburst frequency, as well as depth of modulation), which were significantly reduced after acute spinalization, returned to the control level already on day 3 after spinalization. This rapid increase in activity of F- and E-neurons could be caused by different factors: by spontaneous increase in excitability of spinal interneurons after deprivation of supraspinal influences, by an increase in efficacy of sensory input from limb mechanoreceptors, by an increase in the strength of sensory input (e.g., due to spontaneous increase of excitability of deprived of supraspinal influences gamma-motoneurons leading to an increase in signals from muscle spindles). At the same time point (day 3 after spinalization when activity of F- and E-neurons reached the control level), we found a substantial increase in relative number of residual EMG responses to tilts as compared with that observed after acute spinalization. One can suggest that this increase was caused by restoration of excitability level of spinal interneurons. However, on day 3 EMG responses to tilts in hindlimb extensors were very weak and EMG amplitude constituted only about 12% of control, suggesting that excitability level of motoneurons at this time point remained very low. We found that with time EMG amplitude gradually increased and reached about 50% of control at day 30 after spinalization. Dramatic reduction in excitability level of motoneurons caused by spinalization (primarily due to the loss of persistent inward currents; Conway et al., 1988; Crone et al., 1988; Hounsgaard et al., 1988) and its slow rate of recovery over time was reported earlier (Johnson et al., 2013). Thus, one can conclude that spinalization triggers two processes: fast recovery of excitability level of interneurons (taking days) and slow recovery of excitability level of motoneurons (taking months).

At later time points after spinalization, we found significant changes in some activity parameters of populations of F- and E-neurons located in areas of the gray matter, which were not affected by acute spinalization. This result reflects continuous plastic changes in spinal postural networks taking place with time after spinalization. Most likely, a significant decrease in the depth of modulation of F-neurons located in the dorsal horn on day 30 after spinalization was caused by a significant decrease in the efficacy of sensory input from the ipsilateral limb, while a significant increase in the mean frequency of E-neurons in the ventral horn as well as in intermedial area of the gray matter – by an increase in their excitability level. The reasons for these changes as well as why these changes did not cause any significant changes in motor responses to tilts are not clear.

One of the reasons for abnormal reflex responsiveness characteristic for spasticity is distortions in the processing of sensory information (Frigon and Rossignol, 2006; D’Amico et al., 2014). We found that the processing of tilt-related sensory signals, which was severely distorted after acute spinalization did not recover during following month. Thus, contribution of tilt-related sensory inputs from the ipsilateral and contralateral limbs to modulation of F- and E-neurons did not reach control level within 1 month after spinalization. As after acute spinalization, at each of time points we found an almost twofold decrease in the relative number of neurons with a contribution of input from the contralateral limb (Types 2–4). Most likely, commissural interneurons transmitting signals from the contralateral limb were inactivated by acute spinalization and their excitability level did not reach control level during 1 month of observation. Such commissural neurons, with sensory input from the limb, and inputs from supraspinal structures, have been described (Jankowska, 2008). Recently, contribution of inhibitory V0 commissural interneurons to generation of postural corrections caused by tilts has been demonstrated (Vemula et al., 2018). In addition, we found significant changes in efficacy of tilt-related sensory inputs from ipsilateral and contralateral limb to specific populations of F-neurons taking place at different time points after spinalization. This result is in line with numerous evidences suggesting spontaneous plastic changes in spinal pathways mediating sensory signals from muscle and cutaneous receptors taking place with time after spinalization (Frigon and Rossignol, 2006; D’Amico et al., 2014; Johnson et al., 2017).

Besides the changed relative contribution of sensory inputs from the ipsilateral and contralateral limbs, we observed dramatically modified receptive fields of F- and E-neurons with increased (up to 60% vs. 7% in control and 4% after acute spinalization) relative number of neurons activated from skin/fur receptors. This new sensory input can contribute to recovery of activity value of spinal interneurons after spinalization. Abnormal expansion of receptor fields of motoneurons in spinal subjects was reported earlier (Hyngstrom et al., 2008; Frigon et al., 2011; Johnson et al., 2013).

It was shown that in chronic spinal rabbits, tilts often evoked repetitive EMG bursts superimposed on the ordinary EMG responses (Lyalka et al., 2011). Such EMG bursts (but generated at higher frequencies and termed clonus) are the symptom observed in spinal cord injured patients, and presumably caused by the central generating mechanisms in response to somatosensory signals (Beres-Jones et al., 2003). In rabbits, a gradual increase in oscillatory EMG activity during 1st month after spinalization was reported (Lyalka et al., 2011). It was suggested that this is due to enhancement of excitability in the spinal rhythm-generating networks. In the present study, we found a significant enhancement of the oscillatory activity in F-neurons from day 3 after spinalization, which can contribute to generation of EMG bursting. A gradual increase in EMG bursting during the 1st month after spinalization most likely reflects a slow gradual increase in the excitability level of motoneurons.

One of the symptoms characteristics for chronic spinal subjects is spasms of long duration appeared spontaneously or caused by unspecific sensory stimuli (Brown, 1994; Young, 1994; Hultborn, 2003). Multiple mechanisms underlying generation of spasms were suggested including changes in biophysical properties of motor neurons (Eken et al., 1989; Kiehn and Eken, 1998; Bennett et al., 1999; Li and Bennett, 2003; Li et al., 2004; Murray et al., 2010), reduced presynaptic inhibition of afferents (Faist et al., 1994; Xia and Rymer, 2005), changes in inhibition efficacy (Pierrot-Deseilligny et al., 1979; Shefner et al., 1992; Crone et al., 1994, 2004; Kapitza et al., 2012). A recent study suggests a major role of excitatory (glutamatergic) interneurons in triggering and sustaining the spasms (Bellardita et al., 2017), and in particular, a critical role of V3 interneurons for their initiation (Lin et al., 2019). In the present study, the mechanism underlying generation of spasms was not analyzed, since repetitive sensory stimulation caused by platform tilts abolished spasms.

To conclude, in the present study the activity of spinal neurons of postural networks at different time points after spinalization in mammals have been characterized for the first time. The obtained results suggest that spinalization triggers two processes of plastic changes of postural networks. First, a rapid (taking days) process of recovery of the normal general activity level in spinal interneurons (though with oscillatory activity stronger than in control), which underlies appearance of residual motor responses to posture-related stimuli containing EMG bursting. Second, a slow (taking months) process of recovery of the motoneuronal excitability leading to gradual increase of EMG amplitude of these responses. However, absence of recovery of normal processing of postural sensory signals results in abnormal PLRs and loss of postural functions.

## Data Availability

The datasets generated for this study are available on request to the corresponding author.

## Ethics Statement

Animal Subjects: the animal study was reviewed and approved by Norra Djurförsöksetiska Nämnden in Stockholm.

## Author Contributions

TD and GO designed the experiments. PZ, VL, and TD performed the experiments and analyzed the data. All authors participated in the writing and reviewing of the manuscript.

## Conflict of Interest Statement

The authors declare that the research was conducted in the absence of any commercial or financial relationships that could be construed as a potential conflict of interest.
