# Supplementary material for: Changes in Activity of Spinal Postural Networks at Different Time Points After Spinalization
Source: Front Cell Neurosci. 2019 Aug 21;13:387. doi: 10.3389/fncel.2019.00387 (PMC6712497; doi:10.3389/fncel.2019.00387)
Supplement: Supplementary file 1 [file Image_1.pdf]

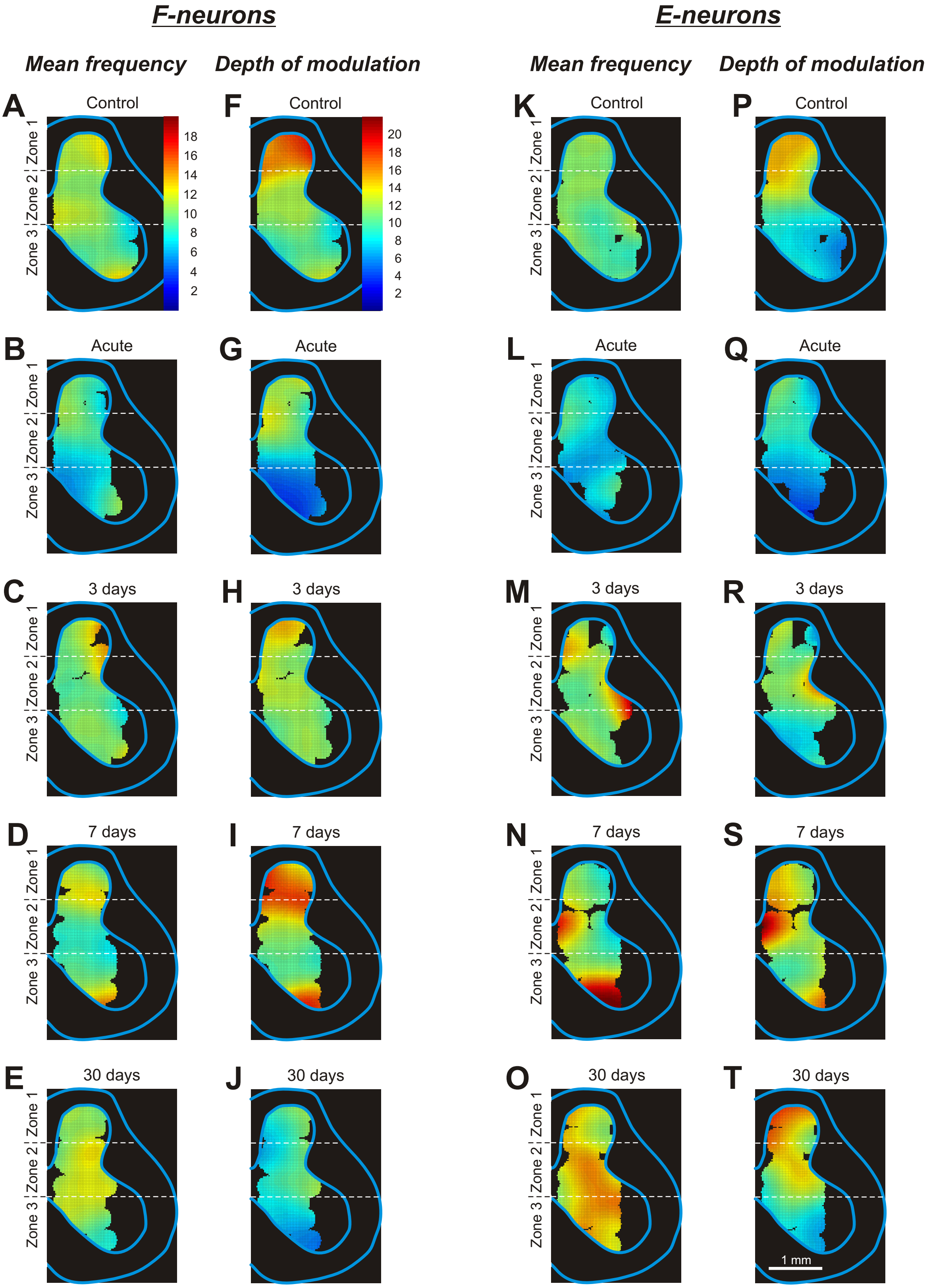

Supplementary Figure S1. The mean frequency and the depth of modulation of local populations of F- and E-neurons during tilts of the whole platform in control and at different time points after spinalization. Averaged distributions of the mean frequency **(A-E, K-O)** and the depth of modulation **(F-J, P-T)** of F-neurons **(A-J)** and E-neurons **(K-T)** on the cross-section of the spinal cord in control **(A,F,K,P)**, after acute spinalization **(B,G,L,Q)**, and on 3rd day **(C,H,M,R)**, 7th day **(D,I,N,S)**, 30th day **(E,J,O,T)** after spinalization. The mean values are presented as heatmaps (see Materials and Methods).
