# Supplementary material for: Changes in Activity of Spinal Postural Networks at Different Time Points After Spinalization
Source: Front Cell Neurosci. 2019 Aug 21;13:387. doi: 10.3389/fncel.2019.00387 (PMC6712497; doi:10.3389/fncel.2019.00387)
Supplement: Supplementary file 2 [file Image_2.pdf]

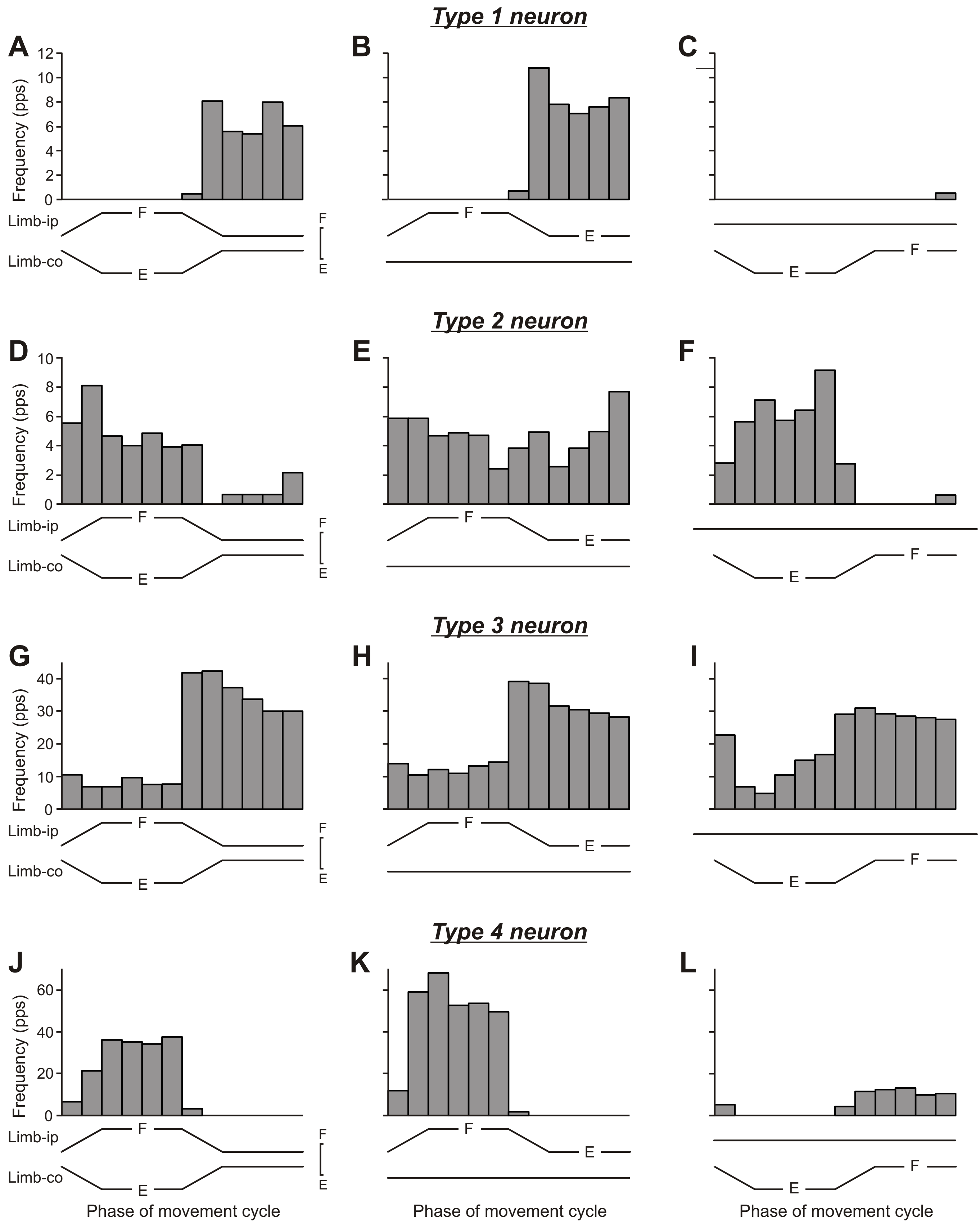

Supplementary Figure S2. Examples of neurons with different sources of modulation recorded at day 3 after spinalization. (A-L) Histograms of activity of individual neurons in the cycle of movement (F, flexion; E, extension) of the ipsilateral limb (Limb-ip) and/or contralateral limb (Limb-co) caused by the whole platform tilts **(A,D,G,J)**, as well as by tilts of its ipsilateral **(B,E,H,K)** and contralateral **(C,F,I,L)** part. Each histogram represents an average of four sequential cycles. **(A-C)** An example of Type 1 neuron that received tilt-related sensory input from ipsilateral limb only. **(D-F)** An example of Type 2 neuron that received tilt-related sensory input from contralateral limb only. **(G-I)** An example of Type 3 neuron that was activated by ipsilateral limb extension and contralateral limb flexion. **(J-L)** An example of Type 4 neuron that was activated by flexion of each of hindlimbs.
